# Neuroprotective effects of açaí (*Euterpe oleracea* Mart.) against diabetic retinopathy

**DOI:** 10.3389/fphar.2023.1143923

**Published:** 2023-04-18

**Authors:** Edwiges de Fátima de Oliveira, Alódia Brasil, Anderson Manoel Herculano, Matheus A. Rosa, Bruno Duarte Gomes, Fernando Allan de Farias Rocha

**Affiliations:** ^1^ Laboratory of Neurophysiology Eduardo Oswaldo Cruz, Institute of Biological Science, Federal University of Pará, Belém, Pará, Brazil; ^2^ Laboratory of Experimental Neuropharmacology, Institute of Biological Science, Federal University of Pará, Belém, Pará, Brazil; ^3^ Faculty of Nutrition, Institute of Health Science, Federal University of Pará, Belém, Pará, Brazil

**Keywords:** *Euterpe oleracea*, oxidative stress, diabetic retinopathy, antioxidant agent, electroretinogram, neuroprotection

## Abstract

**Introduction:** Diabetes mellitus describes a metabolic disorder of multiple etiologies, characterized by chronic hyperglycemia, which induces a series of molecular events capable of leading to microvascular damage, affecting the blood vessels of the retina, causing diabetic retinopathy. Studies indicate that oxidative stress plays a central role in complications involving diabetes. Açaí (*Euterpe oleracea*) has attracted much attention given its antioxidant capacity and potential associated health benefits in preventing oxidative stress, one of the causes of diabetic retinopathy. The objective of this work was to evaluate the possible protective effect of açaí (*E. oleracea*) on the retinal function of mice with induced diabetes, based on full field electroretinogram (ffERG).

**Methods:** We opted for mouse models with induced diabetes by administration of a 2% alloxan aqueous solution and treatment with feed enriched with açaí pulp. The animals were divided into 4 groups: CTR (received commercial ration), DM (received commercial ration), DM + açaí (*E. oleracea*-enriched ration) and CTR + açaí (*E. oleracea*-enriched ration). The ffERG was recorded three times, 30, 45 and 60 days after diabetes induction, under scotopic and photopic conditions to access rod, mixed and cone responses, in addition to monitoring the weight and blood glucose of the animals during the study period. Statistical analysis was performed using the two-way ANOVA test with Tukey’s post-test.

**Results:** Our work obtained satisfactory results with the ffERG responses in diabetic animals treated with açaí, where it was observed that there was no significant decrease in the b wave ffERG amplitude of this group over time when compared to the results of the Diabetic group not treated with açaí, which showed a significant reduction of this ffERG component.

**Discussion:** The results of the present study show, for the first time, that treatment with an açaí-enriched diet is effective against the decrease in the amplitude of visual electrophysiological responses in animals with induced diabetes, which opens a new horizon for the prevention of retinal damage in diabetic individuals from treatment with açaí base. However, it is worth mentioning that our findings consist of a preliminary study and further researches and clinical trials are needed to examine açaí potential as an alternative therapy for diabetic retinopathy.

## 1 Introduction

The pathology known as diabetes mellitus (DM) is within ten main causes of death in Western countries. This disease presents a multiple etiology of metabolic disorder, which induces chronic hyperglycemia and disorders of carbohydrates, proteins, and fats metabolism, a direct result of losses in the secretion of insulin, the action of insulin, or both (Alberti and Zimmet, 1998; Pizova, 2018). The development of DM is composed of several pathogenic processes including the autoimmune destruction of pancreatic cells (DM type 1) or abnormalities that result in resistance to insulin action (DM type 2), in both cases result in inefficient insulin activity in target tissues (Kesavulu et al., 2000).

The chronic hyperglycemia characteristic of DM induces a series of molecular events capable of leading to microvascular damage, affecting the blood vessels of the retina, this is one of the most common complications of DM called diabetic retinopathy (DR) (Khalil, 2017; Wang and Lo, 2018; Ansari et al., 2022). It is known that the predisposition of developing DR is directly proportional to the age of the patient, duration of diabetes, hypertension, and poor glycemic control (Shukla and Tripathy, 2022). According to International Diabetes Federation (IDF) 537 million (10,5%) adults (20–79 years) live with diabetes in 2021 (Sun et al., 2022) and a recent systematic review has concluded that the global prevalence of DR among people with diabetes is estimated at 22.27% (Teo et al., 2021). This is a very worrying data because, in the world panorama, DR is one of the main causes of visual loss and blindness, which generates several disorders to the patient besides the economic impact (Sivakumar et al., 2005; Ozawa et al., 2011; Sivaprasad et al., 2012; Leasher et al., 2016).

Studies indicate that oxidative stress plays a central role in complications involving diabetes (Brownlee, 2001; Reis et al., 2008; Ola et al., 2018; Wang and Lo, 2018; Kang and Yang, 2020; Ansari et al., 2022). The oxidative stress occurs by a serious imbalance between oxidants agents, (which includes reactive oxygen species (ROS) and reactive nitrogen species (NRS)) and endogenous antioxidant (like GSH, glutathione peroxidase, and catalase) (Taniyama and Griendling, 2003; Jones, 2006). Several metabolic pathways are associated with the pathogenesis of DR due to hyperglycemia present in DM, such as glucose auto-oxidation, activation of the polyol pathway, formation of advanced non-enzymatic glycosylation products (AGEs), and hexosamine pathways, developing oxidative stress and inflammation (Maritim et al., 2003; Fong et al., 2004; Ola et al., 2018; Wang and Lo, 2018; Kang and Yang, 2020; Ansari et al., 2022).

The antioxidant and anti-inflammatory activity of some compounds present in foods has aroused scientific interest due to the potential effect on the prevention of tissue injuries caused by oxidative stress, the primary cause of many chronic diseases due to cell damage that, in turn, can promote physiological dysfunctions and cell death, such as diabetic retinopathy in patients with DM. In this context, açaí (*Euterpe oleracea* Mart.), a species belonging to the palm tree (Arecaceae) family, native to countries in the Amazon region of tropical South and Central America, including Brazil, Ecuador, and Venezuela (de Oliveira et al., 2019), has been used in several studies as a potential antioxidant and anti-inflammatory agent that prevents injury to physiological systems (Udani et al., 2011; Gironés-Vilaplana et al., 2014; Brasil et al., 2017; Faria E Souza et al., 2017; da Silva Cristino Cordeiro et al., 2018; de Bem et al., 2018b; De Bem et al., 2018a; Kim et al., 2018; Souza-Monteiro et al., 2019; Magalhães et al., 2020).

Brasil and collaborators observed that animals fed a diet supplemented by açaí did not have neither visual impairment under mercury poisoning, in the vast majority of visual responses assessed, nor MeHg-induced oxidative stress in retinal tissue (Brasil et al., 2017). In another study, it was observed that the use of açaí decreased kidney damage due to the reduction of inflammation, oxidation stress, and improving the renal filtration barrier (da Silva Cristino Cordeiro et al., 2018). De Bem and cols showed that the treatment with açaí associated with physical exercises increases the antioxidant defense and protects diabetic rats against hepatic steatosis (de Bem et al., 2018b).

Furthermore, some studies have shown positive effects of *E. oleracea* attributed to its antioxidant potential on neurodegenerative diseases (de Oliveira et al., 2019; D’Amico et al., 2022), anxiety-like behavior (de Carvalho et al., 2022) and model of aging (Remigante et al., 2022).

Since it is known that diabetes induces oxidative stress and inflammation in the retina leading to neuronal death, flavonoids have provided good results to prevent or treat diabetic retinopathy due to anti-inflammatory, antioxidant properties (Matos et al., 2020). As these are the main bioactive components of açaí and *E. oleracea* has gained prominence in recent years as it has substantially higher antioxidant capacity compared to most dark colored fruits and vegetables (Bichara and Rogez, 2011) we chose this functional food to use in our study.

Several studies had showed positive effects of flavonoids in DR (Ola et al., 2018; Matos et al., 2020) as well as effects of açaí in different models (de Oliveira et al., 2019; D’Amico et al., 2022; de Carvalho et al., 2022; Remigante et al., 2022), including DM models (da Silva Cristino Cordeiro et al., 2018; De Bem et al., 2018a; de Bem et al., 2018b) and protection against retinal electrical response impairment induced by toxic agent (Brasil et al., 2017), however there is no studies testing the acai effect in retinal function in DR model.

In the present study, we tested, for the first time, the hypothesis that açaí acts in the prevention of neurophysiological injuries in the retina of diabetic mice using Electroretinogram (ERG) as a functional integrity measure of the visual system.

## 2 Methods

### 2.1 Animals

The animals used to perform the experiments were adult Swiss mice (25–32 g—male and female) obtained from the Experimentation Laboratory of the Biological Sciences Institute (ICB) of the Federal University of Pará (UFPA). The animals were maintained in polypropylene cages, with a maximum of four animals and 12 h light-dark cycle at 25°C ± 1°C. All experiments were conducted in accordance with the use and care of animals in Ophthalmic and Vision Research (ARVO) and were approved by the Ethics Committee on Experimental Animals of the Federal University of Pará (UFPA). (CEPAE-UFPA: BIO 033.2015).

### 2.2 Diet and experimental groups

The pulp of the pasteurized açaí (*E. oleracea*) was obtained from a local distributor. The diet was elaborated according to the Association of Official Analytical Chemistry (1989) and Reeves et al. (Reeves et al., 1993). The pallets formed of *E. oleracea*-enriched (EO-enriched) rations were made from the commercial ration bran (PURINA Company Cia, São Paulo, Brazil) mixture with the açaí pulp (10:1 g/g), utilizing the ultrapure aqueous medium. The pallets were warmed at 60°C for 3 h and used in all experimental diet procedures and kept under refrigeration ∼4°C, protected from light until the moment of use. The preparation of EO-enriched ration was done at intervals of 3-4 days.

The animals were divided into four groups: Control group 1 (*n* = 4), called CTR, Diabetes group 1 (*n* = 4), called DM–both groups received commercial ration without açaí; Control group 2 (*n* = 4), called CTR + AÇAÍ; and Diabetes group 2 (*n* = 4), called DM + AÇAÍ–both groups received ration enriched with açaí after diabetes induction.

### 2.3 Diabetes induction

The diabetes was induced in the two diabetes groups by administration of 2% alloxan aqueous solution, with a dose of 170 mg/kg of alloxan (Alloxan monohydrate, Sigma-Aldrich, St. Louis, MO, United States) dissolved in 0.2 mL of saline (0.9%) intraperitoneally injected. The animals of two control groups received only 0.2 mL of saline solution. Animals with glycemia above 200 mg/dL (16 mmol/L) were considered diabetic. Confirmation of the diabetes was made 48 h after the induction by an automated process using a glycemic meter (OnCall^®^ Plus, brand ACON Laboratories, Inc.) through blood collection performed with a slight incision in the animal’s tail after fasting previous 12 h. The body mass and blood glucose for all groups were monitored every 15 days and started in the same day of diabetes induction, continuing during the experiment period in all experimental groups.

### 2.4 Electroretinogram (ffERG)

**FIGURE 1:**
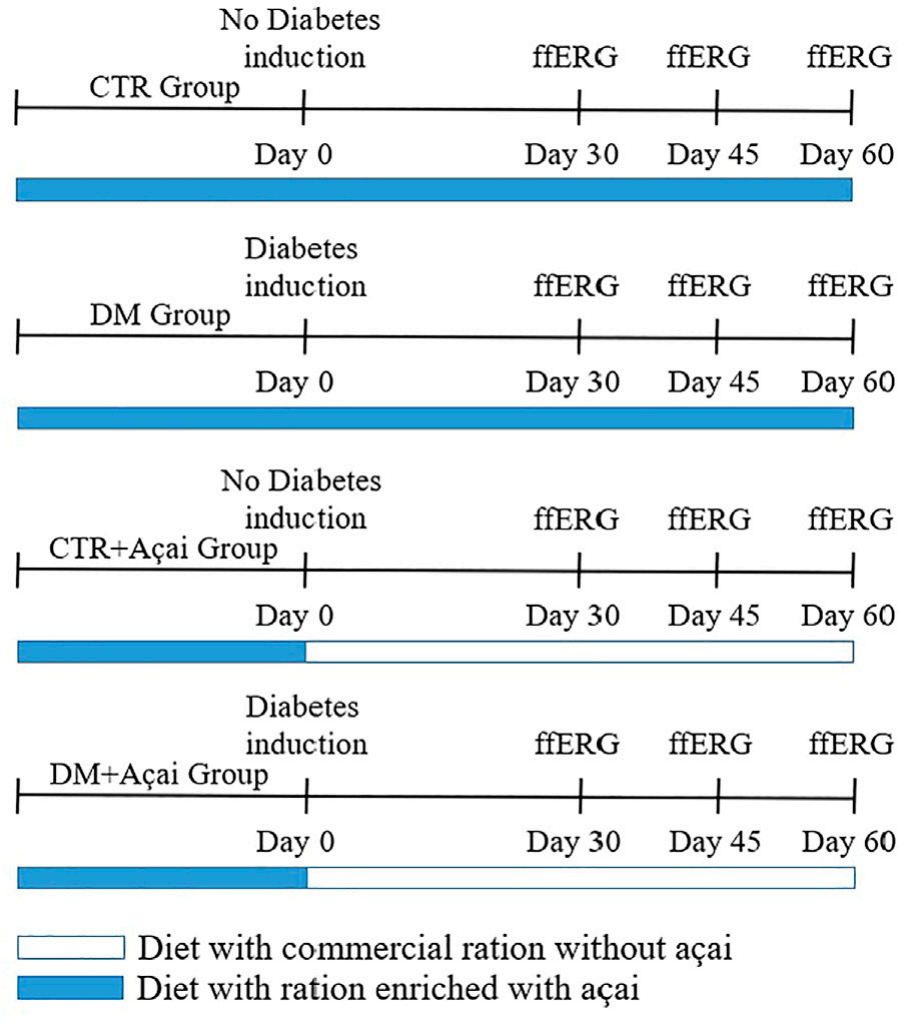
Graphical scheme of study design. The animals were feed with açaí-enriched diet for 28 days and in the next day were subjected to diabetes induction (Day 0). Then, ffERG (full field electroretinogram) was recorded at 30, 45, and 60 days after diabetes induction (Day 30, 45, and 60, respectively).

The full field Electroretinogram (ffERG) was the clinical and laboratory electro functional test chosen to evaluate a retinal function of the experimental groups. Electroretinographic experiments were records three times, 30, 45, and 60 days after the induction of diabetes. The study design can be seen in Figure 1. The animals were adapted to the dark for at least 14 h (“overnight”) in the experiment room. The animals were anesthetized with an intraperitoneal injection of ketamine hydrochloride solution (100 mg/kg)/xylazine hydrochloride (6 mg/kg), the pupils were dilated with a drop of 1% tropicamide eye drops (Mydriacyl Alcon®), the animals them were transferred to a Faraday cage.

The ffERG records are obtained through the electrodes, positioned as follows: active silver-filament electrode positioned on the cornea with 2% methylcellulose eye drops to protect the surface of the cornea; a subcutaneous steel needle electrode in the eyelid was used as the reference electrode (model F-E3-48, Grass instruments, Warwick, United States); and a Gold *disk* skin electrode placed in the ear of the animal served as the ground electrode (model F-E6SHC-12) fixed by electrolytic paste (Ten20, DO WEAVER) applied to the electrode, to facilitate electric conduction. The procedure is done with the animals already positioned in the Faraday cage (46.5 × 40.3 × 30 cm), 30 cm from the photo stimulator. These procedures were performed in the dark with the assistance of a low-intensity red light.

The light stimuli were presented by photo-stimulator (Model PS33-PLUS, Grass Technologies, Warwick, United States) with different intensities, duration, and interval. The stimuli protocol was based on the standards established by Harazny et al. (Harazny et al., 2009).

The rod-driven response was obtained in an adaptation condition during the overnight period with stimulus intensity at ∼0.09 cd∙s∙m−2. In addition to the isolated rod-driven response, it was the measure of the combined rod-cone responses, called the mixed 1, under stimulation at 0.378 cd∙s∙m−2, and mixed 2, at 10.215 cd∙s∙m−2. After recording the scotopic responses, the animals were light-adapted for 10 min to obtain photopic cone single flash responses stimulated by flashes of 10.215 cd∙s∙m−2.

The intervals between stimuli for the responses in scotopic conditions were 15 s and responses under photopic conditions, was 1 s. Light-evoked responses from retinal tissue were amplified 50,000 x with a AC amplifier (Model P511, Grass Technologies, Warwick, United States), filtered between 0.3 and 300 Hz and digitalized with an analog-digital interface (National Instruments, Austin, TX). The data acquisition program used was Labview 3.0 (National Instruments, Austin, TX).

In the present work, the ffERG component analyzed was only the b-wave amplitude (positive ERG component generated in inner nuclear retinal layer, reflecting bipolar ON cells and Müller cells activity), which was measured from the trough of the a-wave or from the baseline to the peak of the b-wave and expressed in microvolt (µV).

### 2.5 Statistical analysis

Results were submitted to statistical analysis using the program GraphPad Prism 5, where the mean and the standard deviation of the experimental groups were determined. The data were, then, compared by the one-way ANOVA followed by *Tukey* post-tests. Values of *p* < 0.05 were considered statistically significant.

## 3 Results

### 3.1 Body mass and glycemia monitoring

**FIGURE 2:**
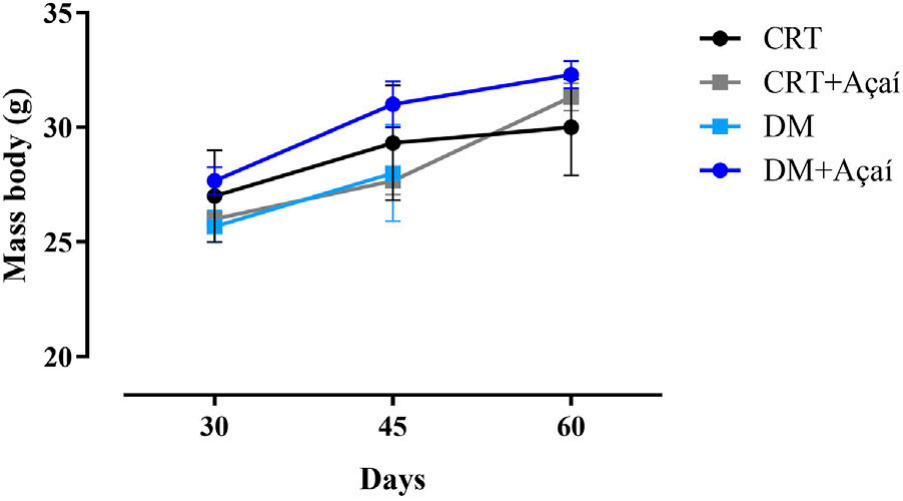
Mass body (g) monitoring after diabetes induction. Values are means ± SD. At 30, 45, and 60 days after diabetes induction, there was no statistical difference in the comparison of body mass between groups (*p* > 0.05).

**FIGURE 3:**
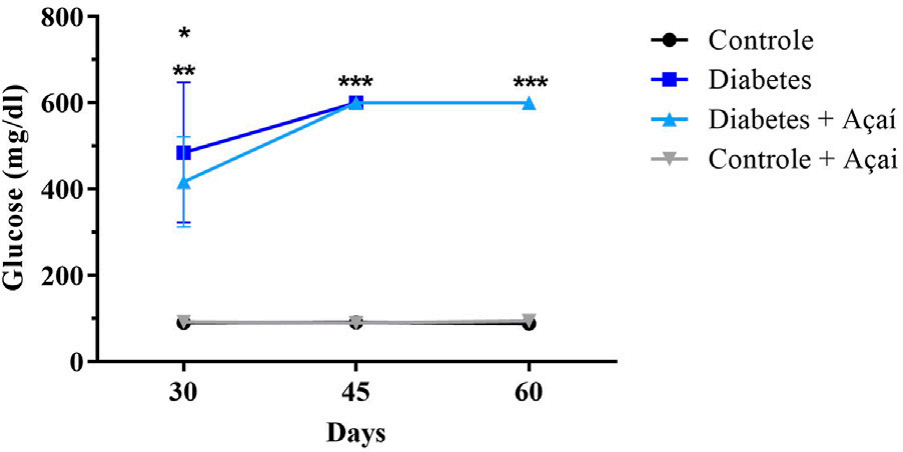
Blood glucose levels (mg/dL) monitoring after diabetes induction. Values are means ± SD. ** *p* < 0.001 CTR and CTR + Açaí vs. DM in 30 days, * *p* < 0.01 CTR and CTR + Açaí vs. DM + Açaí in 30 days, * *p* < 0.0001 CTR and CTR + Açaí vs. DM and DM + Açaí in 45 days, * *p* < 0.001 CTR and CTR + Açaí vs. DM + Açaí in 60 days.

Body mass and blood glucose are shown at 30, 45, and 60 days after diabetes induction (Figures 2, 3). However, the diabetic group could only be followed up to 45 days after induction; this group did not survive up to 60 days. All groups showed an increase in body mass over time (Figure 2). At 30 days, the mean body weight of the CTR group was 27 g, the CTR + Açaí group 27.67 g, the DM group 25.67 g, the DM + Açaí group 26 g, with no statistical difference (*p* > 0.05). In 45 days, the DM and CTR + Açaí groups had the lowest mean body weight compared to the other groups, but without statistical difference (*p* > 0.05). In addition, in 60 days, the CTR, CTR + Açaí and DM + Açaí groups again presented similar weight 30, 32.3, and 31.33 g, respectively, and without statistical difference (*p* > 0.05).

The blood glucose monitoring showed that CTR and CTR + Açaí group remained of blood glucose levels about 100 mg/dL, whereas in the DM group and DM + Açaí remained the mice presented mean values ranging from 400 to ≥600 mg/dL over the days after diabetes induction (Figure 3). Thereby, DM and DM + Açaí groups did not differ from each other but presented blood glucose higher than CTR and CTR + Açaí groups, confirming the induction of diabetes.

### 3.2 Full field electroretinogram (ffERG)

In Table 1 we have compiled all responses of medium amplitude. The amplitudes refer to the b-wave responses of each applied protocol.

**TABLE 1:** Amplitudes responses compilation from b-wave by all ERG protocols.

b-wave amplitude of scotopic response												
	CTR			CTR + açaí			DM			DM + açaí		
Days	Mean	SD	*N*	Mean	SD	*n*	Mean	SD	*n*	Mean	SD	*n*
30	202.41	7.96	4	200.19	22.74	4	149.54	12.78	4	182.87	11.24	4
45	188.19	19.81	4	199.26	20.76	4	69.72	13.28	4	179.04	8.47	4
60	174.68	15.00	4	191.49	10.62	4	—	—	—	164.35	24.30	4
b-wave amplitude of combined response (mix 1)												
	CTR			CTR + Açaí			DM			DM + Açaí		
Days	Mean	SD	*N*	Mean	SD	*n*	Mean	SD	*n*	Mean	SD	*n*
30	247.96	12.03	4	242.07	34.00	4	141.47	9.09	4	244.19	16.34	4
45	212.89	11.30	4	245.66	11.00	4	62.23	10.72	4	198.74	24.30	4
60	143.73	12.41	4	143.63	5.00	4	—	—	—	124.80	18.10	4
b-wave amplitude of combined response (mix 2)												
	CTR			CTR + Açaí			DM			DM + Açaí		
Days	Mean	SD	*n*	Mean	SD	*n*	Mean	SD	*n*	Mean	SD	*n*
30	293.16	4.43	4	312.52	13.45	4	194.82	14.00	4	284.64	9.33	4
45	259.25	15.10	4	270.01	20.01	4	168.47	9.54	4	230.76	18.34	4
60	252.70	14.34	4	259.92	17.54	4	—	—	—	215.38	22.50	4
b-wave amplitude of cone response												
	CTR			CTR + Açaí			DM			DM + Açaí		
Days	Mean	SD	*n*	Mean	SD	*n*	Mean	SD	*n*	Mean	SD	*n*
30	270.35	11.86	4	263.06	12.85	4	181.47	8.66	4	245.88	13.39	4
45	217.22	14.97	4	231.71	22.51	4	127.07	9.54	4	209.23	17.53	4
60	192.36	5.14	4	194.24	5.00	4	—	—	—	170.84	12.27	4

#### 3.2.1 Scotopic response (rods)

**FIGURE 4:**
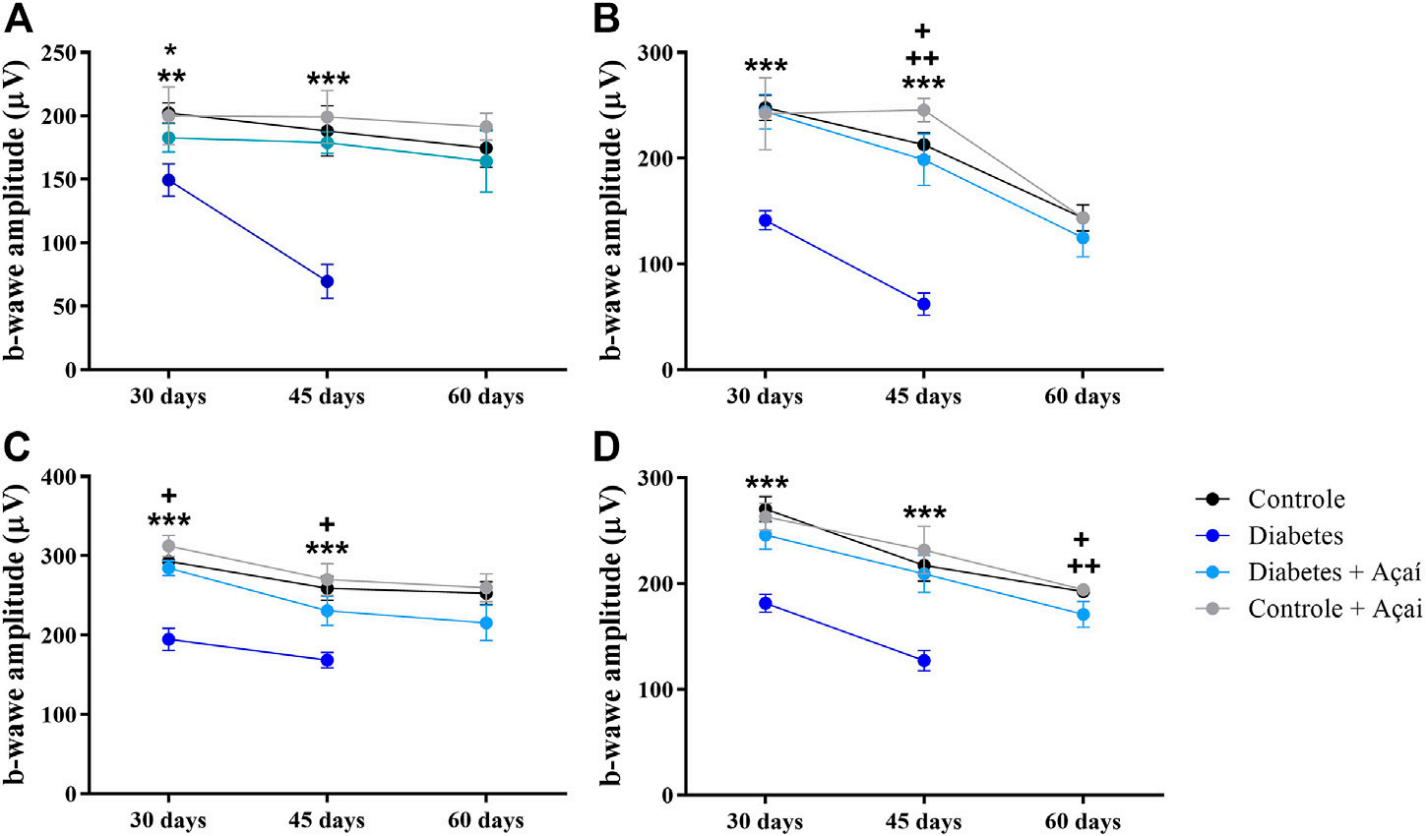
Full field electroretinogram b-wave amplitude s monitoring after diabetes induction. Values are means ± SD. **(A)** Scotopic response (Rods): ** *p* < 0.001 CTR and CTR + Açaí vs. DM in 30 days; * *p* < 0.01 DM + Açaí vs. DM in 30 days; *** *p* < 0.0001 CTR, CTR + Açaí and DM + Açaí vs. DM in 45 days; + *p* < 0.01 CTR + Açaí vs. CRT and DM + Açaí in 45 days **(B)** Scotopic combined response (Mix 1): *** *p* < 0.0001 CTR, CTR + Açaí and DM + Açaí vs. DM in 30 days; *** *p* < 0.0001 CTR, CTR + Açaí and DM + Açaí vs. DM in 45 days; + *p* < 0.01 CRT vs. CTR + Açaí in 45 days; ++ *p* < 0.01 DM + Açaí vs. CTR + Açaí in 45 days **(C)** Scotopic combined response (Mix 2): *** *p* < 0.0001 CTR, CTR + Açaí and DM + Açaí vs. DM in 30 days; + *p* < 0.01 DM + Açaí vs. CTR + Açaí in 30 days; *** *p* < 0.0001 CTR, CTR + Açaí and DM + Açaí vs. DM in 45 days; + *p* < 0.01 DM + Açaí vs. CTR + Açaí in 45 days **(D)** Cone response: *** *p* < 0.0001 CTR, CTR + Açaí and DM + Açaí vs. DM in 30 days; *** *p* < 0.0001 CTR, CTR + Açaí and DM + Açaí vs. DM in 45 days; + *p* < 0.01 CRT vs. CTR + Açaí in 60 days; ++ *p* < 0.01 CTR + Açaí vs. DM + Açaí in 60 days.

Under dark adaptation, the amplitude of rod-driven responses decreased significantly in DM animals within the first 30 days after diabetes induction, mean 149.54 μV (±12.78, *p* < 0.001) (Figure 4A). On the other hand, the DM + Açaí animals had a mean response amplitude of 182.87 μV (±11.24) similar and without significant difference to the CRT 202.41 ± 7.96 μV and CRT + Açaí 200.19 ± 22.74 μV groups (*p* > 0.05). At Day 45, there was a greater decrease in the response amplitude of the DM group (69.72 ± 13.28 μV, *p* < 0.001), in relation to the CRT, CRT + Açaí and DM + Açaí groups, which maintained similar responses and without significant difference (*p* > 0.05), 188.19 ± 19.81 μV, 199.26 ± 20.76 μV, 179.04 ± 8.47 μV, respectively. At Day 60, the CRT, CRT + Açaí and DM + Açaí groups maintained similar average response amplitudes and without significant difference between the groups 174.68 ± 15 μV, 191.49 ± 10.62 μV, 164.35 ± 24.3 μV, respectively (*p* > 0.05). Unfortunately, at 60 days, the diabetic animals did not resist, and we did not have amplitude measurements for this group.

#### 3.2.2 Scotopic combined response (rods and cones)

In the protocols of combined responses from rods and cones (Mix 1 and Mix 2) we obtained a very similar behavior of the responses when we tested just rods. Here, DM group had a significant reduction in the average amplitude of the b-wave responses comparing with the other groups at 30 days after diabetes induction in the Mix1 (DM = 141.47 ± 9.09 μV, *p* < 0.0001) and Mix2 (DM = 194.82 ± 14.00 μV, *p* < 0.0001) protocol (Figures 4B, 4C). In the same period, the DM + Açaí group maintained responses without statistical difference for the CTR group (*p* > 0.05), however, the CRT + Açaí group showed significantly greater responses than the CRT and DM + acai groups in the Mix2 protocol in 30 days (*p* < 0.01). In 45 days, the DM group, in both protocols, again presented a decrease in response amplitude 62.23 ± 10.72 μV (*p* < 0.0001) in the Mix1 protocol and 168.47 ± 9.53 μV (*p* < 0.0001) in the Mix2 protocol, these responses were significantly lower than the amplitudes recorded by the CRT, CRT + Açaí and DM + Acaí groups. At 45 days, we also observed amplitudes of ERG response between the CRT and DM + açaí groups did not differ between each other (*p* > 0.05) but they had amplitudes statistically smaller than the amplitudes of the responses of the CRT + Açaí group in both protocols (Figures 4B, 4C). At 60 days, we noticed amplitudes with no significant difference between the CRT and DM + açaí and DM + açaí groups in mix1 or mix2 protocol (*p* > 0.05).

#### 3.2.3 Photopic response (cones)

In the isolated responses of cones 30 days after the induction of diabetes, the CRT group had an average amplitude of 270.35 ± 11.86 μV, with no statistical difference for the average amplitude of the DM + açaí group 245.88 ± 13.39 μV and CRT + açaí 263.06 ± 12.85 μV (*p* > 0.05). In contrast, the DM group (181.47 ± 8.66 μV, *p* < 0.0001) had a significantly lower ERG response amplitude compared to the other groups. As described in previously protocols in this paper, the responses of the cone system on the ERG had reduced amplitude in 45 days in the DM group, 127.07 ± 9.54 μV comparing with the other groups (*p* < 0.0001). During this period, the mean cone responses were 217.22 ± 4.97 μV for the CRT group, 209.23 ± 17.53 μV for the DM + açaí group and 231.71 ± 22.51 μV for the CRT + açaí group, with no statistical difference between these three groups (*p* > 0.05). At 60 days, we found a statistical difference in response amplitude between the DM + Açaí group vs. the CRT (*p* < 0.01) and CRT + Açaí (*p* < 0.01) groups (Figure 4D), with a lower amplitude in the DM + Açaí group.

## 4 Discussion

The results of our work show that treatment with açaí-enriched diet was effective against the decrease in b-wave amplitude in mice with induced diabetes (DM + Açaí group), preventing diabetes from impairing the functions of retinal cells reflected in the attenuated responses of the ERG in the animals of the DM group. Our data mainly showed that the retinal functional responses of diabetic animals that received açaí-enriched diet did not statistically differ from the responses of the control groups in almost all protocols tested (except in cone response at day 60), these results demonstrate a potential neuroprotective effect of açaí against the damage promoted by diabetes in the retina, reflecting on the preservation of retinal function. The present result about mass body corroborates the previous studies carried out by our group using a similar fortified feed that showed that EO-enriched diet did not influence the animals mass body (Brasil et al., 2017; de Carvalho et al., 2022).

Regarding the analysis of the glycemia of the animals, the control group that received diet supplemented with açaí did not have an increase in glycemia throughout the study period, which can be clarified by Souza et al. (Oliveira de Souza et al., 2010), who in their work showed that the açaí pulp has a low sugar content and is not considered a rich source of carbohydrates, not causing possible harm to the group and should not cause an increase in the glycemic rate of the CTR + Açaí group. Our results of blood glucose level corroborate the study of da Silva Cristino Cordeiro et al. (2018) that fund no difference between the group of rats with streptozotocin (STZ)-induced diabetes and those streptozotocin (STZ)-induced diabetes treated daily with *E. oleracea Mart*. seed extract (200 mg/kg per day, in drinking water), for 45 days (da Silva Cristino Cordeiro et al., 2018).

On the other hand, this result is in disagreement with some studies that show an antidiabetic effect with açaí-induced blood glucose reduction, probably due to differences in experimental design, such as the part of the plant used, form of apresentation/prodution (açaí seed extract, for example, instead of pulp), route of administration and treatment duration (de Oliveira et al., 2015; De Bem et al., 2018a). The lack of effect of the experimental diet on blood glucose shows that the beneficial effect on retinal function is not related to the control of hyperglycemia.

We must highlight the fact that the animals in the DM group did not survive up to 60 days of treatment, in contrast to the DM + Açaí group, which remained alive throughout the test period. Despite proving our hypothesis that açaí acts as a neuroprotector against the deleterious effects of diabetes on the visual system, the fact that diabetic animals treated with açaí remained alive after 60 days was very curious and shows that even at “toxic” doses of alloxan animals treated with açaí remained alive for a long time. This result shows a protective effect of açaí that goes beyond the visual system and that should be investigated in further studies.

With the analysis of the records obtained by the ERG, it was possible to observe the decrease in the amplitude of the b wave of the diabetes group in all types of responses evaluated (scotopic stick, mixed scotopic of 1 and 2 and photopic) in 30 and 45 days. The amplitude most affected was the mixed scotopic response 1 (which is related to the rod and cone response), resulting in a loss of about 43% at 30 days and 71% at 45 days after the induction of diabetes, resulting in a decrease in wave b in relation to the CRT, CRT + Açaí and DM + Açaí groups.

In our study, induced diabetic retinopathy affected the responses of cones and rods, as there was a reduction in the amplitude of the b wave under conditions of scotopic and photopic adaptation, indicating deficits in the different retinal cells responsible for this component (bipolar ON cells and Müller cells). These data were also compatible with the data obtained by Abdelkader (Abdelkader, 2013) in ERG with humans, where changes in scotopic and photopic responses were obtained, and Kohzaki et al. (Kohzaki et al., 2008) reporting a decrease in b wave amplitude.

The data from this work could demonstrate the maintenance of the amplitude of the b wave of the animals that received the diet enriched with açaí in most of the ERG responses, possibly due to its antioxidant and anti-inflammatory effect caused by the açaí in the retina of the mice with diabetes, since these two pathways are the main ones involved in DR. Such effects may be related to the presence of flavonoids, especially anthocyanins, in açaí. Corroborating a similar work carried out by (Ozawa et al. (Ozawa et al., 2011), where ERG impairment due to oxidative stress in mouse retinal neuronal cells is suppressed due to antioxidant administration, which suppresses local ROS.

Brasil et al. (2017) demonstrated significant results using a diet enriched with açaí as a prevention of the impairment caused by MeHg intoxication in the ERG of rats, they also shown an oxidative damage in retinal tissue MeHg-induced and prevented by açaí supplementation, suggesting this pathway as possible cause of preservation of retinal function (Brasil et al., 2017).

Acquaviva et al. (2003) demonstrated that anthocyanins cyanidin-3-glucoside and cyanidin-3-rutinoside had a protective effect on DNA, showed dose-dependent free radical scavenging activity, and significantly inhibited xanthine oxidase activity (Acquaviva et al., 2003). These anthocyanins were the most predominant in the analysis of açaí pulp (Pacheco-Palencia et al., 2009).

Some studies show positive effects of açaí on neurodegenerative diseases which have oxidative stress and neuroinflammation as their basis (de Oliveira et al., 2019). D'Amico et al. (2022) showed that orally supplementation with Açaí berry (500 mg/kg) in an experimental models of Parkinson’s Disease (PD) was able to mitigates PD progression reducing motor and non-motor symptom and neuronal cell death of the dopaminergic tract. The authors attribute the found effects probably to the bioactive components with antioxidant and anti-inflammatory functions. ALNasser et al. (2022) suggest açaí berry as a potential natural treatment for Alzheimer’s Disease (AD), once they showed anti-cholinesterase and antioxidant capabilities of the aqueous and ethanolic extracts. Thereby, açaí berry may limit the pathological deficits found in AD (ALNasser et al., 2022).

In addition, recently our group showed that *E. oleracea*-enriched diet provided anxiolytic-like effects and improves memory consolidation in rats, probably due to the reduced levels of lipid peroxidation (markers of oxidative damage) in the hippocampus (de Carvalho et al., 2022).

Açaí extract also presented beneficial effects against Age-Related Oxidative Stress in a d-Gal-induced model of aging in human erythrocytes. Since aging of these cells is related to oxidative stress and *E. oleracea* presents antioxidante properties, this functional food could improve the functions of erythrocytes and, consequently, the homeostasis of the organism as a whole, counteracting age-related changes (Remigante et al., 2022).

The present work demonstrated, for the first time, that a diet enriched with *E. oleracea* fruit pulp is able to prevent electrophysiological impairment induced by diabetic retinopathy. However other benefits of flavonoids in diabetic retinopathy are already known (Ola et al., 2018; Matos et al., 2020).

Experimental studies have shown that dietary flavonoids induce reduction in oxidative stress in diabetic retina. Among the most studied to improve retinal damage are the flavonoid families: flavanones, flavanols, flavonols, isoflavones, flavones and anthocyanins. Their known antioxidants effects may improve the retinal degenerative factors in diabetes by ameliorating the altered levels of neurodegenerative factors, angiogenic factors, preventing the disruption of the blood–retinal barrier (BRB), decrease the release of proinflammatory mediators, improve the oxidative state, prevent the reduction in retina thickness by attenuating apoptosis and neurodegeneration, and to ameliorate the level of neurotrophic factors which are necessary for the maintenance of neuronal retina (Ola et al., 2018; Matos et al., 2020). Clinical studies have shown that consumption of flavonoids, in both diet or supplements, exerts beneficial effects at different stages: preventing the onset of diabetes, the development of Diabetic Retinopathy in diabetics and prevent the worsening of Diabetic Retinopathy (Matos et al., 2020).

Therefore, intake of foods rich in flavonoids such as açaí would limit oxidative stress and thus prevent neurovascular damage, visual function impairment, and consequently the development or worsening of Diabetic Retinopathy.

## 5 Conclusion

In conclusion, diabetes provoked b-wave amplitude decreases in all the ERG protocols tested, rod, mixed and cone responses at 30 and 45 days after induction of diabetes, while the açaí-enriched diet was able to prevent electrophysiological impairment induced by diabetic retinopathy in all almost the protocols. Furthermore, the *E. oleracea*-enriched diet did not affect the animals nor prevent diabetes-induced hyperglycemia. However, it is worth mentioning that it is a preliminary study and further researches and clinical trials are needed to examine açaí potential as an alternative therapy for diabetic retinopathy.

## Data Availability

The raw data supporting the conclusion of this article will be made available by the authors, without undue reservation.
